# Neutron and capture gamma along the mazes of linear accelerator vaults

**DOI:** 10.1120/jacmp.v4i2.2532

**Published:** 2003-03-01

**Authors:** Raymond K. Wu, Patton H. McGinley

**Affiliations:** ^1^ Department of Radiation Oncology & Biophysics Eastern Virginia Medical School 600 Gresham Drive Norfolk Virginia 23507; ^2^ Division of Radiation Therapy The Emory Clinic 1365 Clifton Road Atlanta Georgia 30322

**Keywords:** neutron dose, capture gamma, shielding, maze

## Abstract

Neutron and photon dose equivalents at various points along the mazes of the vaults for two 15 MV linear accelerators were measured. The measurements were made with the machines set at various gantry angles with and without the scattering phantom, and with the collimators set at the maximum and the minimum field sizes. Neutron dose equivalent measurements were made for five other accelerator vaults. Empirical equations were used to fit the dose data at points along the center of the maze, at 1 m above floor level, with the primary radiation beam pointing downward. It is reported here that both the capture gamma and the neutron dose attenuations along the maze are in agreement with the literature. The neutron dose is dependent on the square root of the ratio of the cross‐sectional areas of the inner maze entrance and the maze. The tenth value distance $\left(T_{N}\right)$ is proportional to the square root of the cross‐sectional area of the maze.

PACS number(s): 87.52.Ga

## INTRODUCTION

The production of neutron and associated capture gamma becomes significant when the energy of a linear accelerator is higher than 10 MV. A frequently used design of a high energy linear accelerator vault includes a main treatment room with a maze leading to the vault entrance. In addition to the maze wall shielding the direct neutron flux, the maze length allows the use of a much lighter door for the maze entrance. Methods used in estimating the neutron dose equivalent at the external maze entrance of medical accelerator vaults are available in the literature.^1^ ^,^ ^2^ However, the methods are based on shorter mazes, and the uncertainty in extending the methods for use in longer mazes is unknown. In addition, the methods do not take into consideration the maze cross‐section and the area of the inner maze entrance, which are known to impact on the neutron dose and tenth value length of its attenuation along the maze.^3^ ^,^ ^4^ In this work, the neutron and photon dose equivalents were measured along the mazes for several accelerator vaults with maze lengths up to 9 m. The measured data for photon dose were fitted to an empirical equation relating the dose to the total neutron fluence at the inner maze entrance, and the distance along the maze. The neutron data were fitted to an empirical equation relating the dose equivalents to the cross‐sectional areas of the maze and the inner maze entrance, the total neutron fluence at the inner maze entrance, and the distance along the maze. An empirical equation was also obtained relating the tenth value length of neutron dose reduction along the maze to the cross‐sectional area of the maze.

## MATERIALS AND METHODS

The first set of measurements was made along the maze centerline of a recently constructed vault for a medical linear accelerator (Varian 2100EX, Varian Medical Systems, Palo Alto, CA).

**Figure 1:**
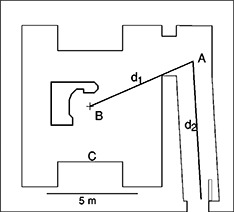
Concrete vault layout showing the point *A*, which is on the centerline of the maze just visible from the isocenter, point *B.*

The manufacturer stated photon energies of the dual energy machine are 6 and 15 MV. Only the results for the 15 MV modality are presented in this work. The 100 cm SSD 10 cm×10 cm field has a percent depth dose value of 76.4%. The ionization ratio is 0.76 according to the TG21 protocol,^5^ and the nominal accelerating potential (NAP) is 12.9 MV The vault was constructed of steel reinforced concrete. The concrete dry density was 2350 kg $m^{-3}$. The layout of the vault is shown to scale in Fig. 1. The accelerator rotation axis is as shown.

The photon measurements were made using an ion chamber type survey meter, Inovision/Victoreen model 450P (Inovision Radiation Measurements, Cleveland, OH). The survey meter was calibrated, and the factor traceable to NIST by K & S Associates (Nashville, TN) within one year prior to and after the work. The neutron response of a survey meter of the same model has been shown to be negligible by McGinley and Miner.^6^

The neutron dose equivalent measurements were made using an Inovision Model 190N meter. The neutron meter is based upon the standard Anderson and Braun design, with a ${\text{BF}}_{3}$ proportional counter operating at 1150 V. The active length is 5.08 cm. The gamma and x‐ray rejection was tested in fields of ${}^{137}\text{Cs}$ and 0.5 MeV scatter up to $5\;\text{Sv}-h^{-1}$. The neutron meter was calibrated by the manufacturer before and after this work. At calibration, the meter was oriented with the specific side of the long axis perpendicular to the general direction of the neutron source. The calibration certificate stated, “Verification of this instrument has been performed using Ra‐Be fast neutrons. The radiation rate measured from the Ra‐Be neutron source was calibrated by using a transfer standard calibrated to a NIST traceable Cf‐252 neutron source. The expanded uncertainty of the calibration is 23%. This value was determined using NIST Technical Note 1297 and the ISO Guide to the Expression of Uncertainty in Measurement (1993).”

The neutron meter showed a significant variation in the isotropy test, in which the meter was rotated horizontally at 45° intervals and readings were taken. Data reported in this work obtained with the axis of the meter perpendicular to the general direction of neutrons as at calibration are denoted as nominal, or as measured at “nominal” angle.

Vault‐2 measurements were made around the same time using the same instruments described above. The two vaults are adjacent to each other, and are very similar in layout and construction. The accelerator (Varian Clinac 1800) has photon energies of 6 and 15 MV Only the data for 15 MV are presented here. The TG21 NAP is the same as the machine in Vault‐1.

The second set of measurements was made for five medical linear accelerators of energies 15 to 25 MV, housed by five different vaults with similar maze design but with different maze lengths, maze cross‐sectional areas, and inner maze entrance areas. Only neutron dose measurements are presented here. The measurements were made using a neutron survey meter made by

Eberline (Eberline Instruments, Santa Fe, NM). The model of the reader was ESP‐1, and the moderator was the NRD neutron REM detector, a 23 cm diameter polyethylene sphere with a ${\text{BF}}_{3}$ tube in the center. The meter has a current NIST traceable calibration.

The two neutron meters were intercompared and the result showed agreements well within the calibration uncertainty quoted above. Some readings presented in this work were obtained using both sets of meters. The differences are well within 10%.

All measurements were made with the meter placed on top of a wood stand at 1 m above the floor. The door at the maze entrance was kept opened during the measurements. The photon and neutron dose data were normalized to isocenter dose (Gy) for 10 cm×10 cm field. The isocenter dose was calibrated to water according to the TG51 protocol^7^ at dose maximum depth at 100 cm SSD. Both the neutron and photon dose data were obtained with the meters set at the integrating mode. For each data point, 2 to 4 Gy was given to the isocenter. The accelerator output dose rate was set at $3-5\;\text{Gy}-{\text{min}}^{‐1}$.

The room and maze dimensions and other parameters for all seven vaults are tabulated in Table I. The first data point along the maze was taken at point *A* for all seven vaults. Point *A* is the point at 1 m above floor, on the centerline of the maze, just visible from the isocenter. (See Fig. 1.) All other data points were obtained at 0.5 to 1 m intervals along a straight line joining point *A* and a point near the maze entrance on the maze centerline.

**Table I:** Room and maze dimensions and other parameters of the seven vaults in this study. NAP is the nominal accelerating potential defined in the TG‐21 protocol.^5^ $S_{1}$ is the product of maze height $\left(h_{1}\right)$ and maze width $(w_{1}).S_{0}$ is the product of inner maze entrance height $\left(h_{0}\right)$ and width $\left(w_{0}\right)$, determined parallel to the end of the maze wall. The value for Vault‐5 is exceptionally low because the vault is a modified betatron room, and the air handling duct work does not penetrate above this entrance as usually the case. $T_{N}$ is the tenth value length, equal to $2.06\times \sqrt{S^{1}}$ (see text). $Q_{N}$ is the apparent neutron source strength (see text). $d_{1}$ is the distance from isocenter to point A as shown in Fig. 1. S is the surface area of the room, excluding the maze. $d_{2}$ max is the distance from point A to the maze entrance.

Vault No.	Vault‐1	Vault‐2	Vault‐3	Vault‐4	Vault‐5	Vault‐6	Vault‐7
Make	Varian	Varian	Philips	Varian	Varian	Varian	Siemens
Model	2100EX	1800	SL25	2300CD	2300CD	2100C	MD‐2
Stated MV	15	15	25	20	18	18	15
NAP MV	12.9	12.9	22	18.5	16.8	16.8	13.2
$Q_{N}1E+12$	0.5	0.5	2.37	1.22	1.22	1.22	0.19
Maze $h_{1}(m)$	4.17	4	3.8	4.04	4.58	4.01	3.51
Maze $w_{1}(m)$	2.1	2.35	1.98	2.62	1.78	1.9	2.13
$S_{1}(m^{2})$	8.76	9.4	7.52	10.6	8.15	7.62	7.48
$T_{N}$ (m)	6.1	6.32	5.65	6.7	5.88	5.69	5.63
Inner $h_{0}(m)$	4.17	4	3.8	4.04	2.13	3.66	3.51
Inner $w_{0}(m)$	2.45	2.45	2.44	3.25	1.22	1.88	2.34
$S_{0}(m^{2})$	10.2	9.8	9.27	13.1	2.6	6.88	8.21
$d_{1}$ (m)	6.3	5.6	6	7	6.06	6.34	5.93
Area *S* (m^2^)	234	189	265	248	220	196	195
$d_{2}$ max (m)	9	6	6	7	8.2	6	5

## RESULTS

### A. Capture gamma

**Figure 2:**
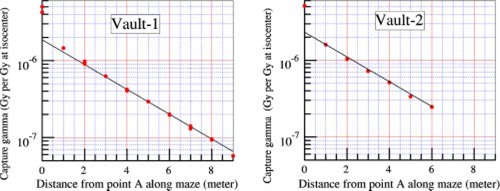
(Color) Measured photon dose along the maze for the two 15 MV accelerators, with beams pointing downward, collimators set to $0\times 0\;{\text{cm}}^{2}$. Calculated values based on Eq. (1) are in solid lines.

The photon dose at any point in the maze is mainly attributed to capture gamma, scatter from patient or phantom, scatter by walls, and radiation transmission through the maze wall. The scatter and leakage radiation doses can be calculated using methods available in the literature.^2^ ^,^ ^8^ Based on these methods it is expected that these components diminish as the distance from point *A* increases. The thicknesses of the maze walls for both vaults are sufficient such that the radiation transmitted through the maze wall is negligible. Capture gamma is the product of neutron capture where the compound nucleus is raised to an excited state and then discharge its energy through the emission of a gamma ray photon. In this section of the study, since the capture gamma dose is the focus of the work, the field size was collimated to 0 cm, so that the scatter components were also kept to the minimum. Figure 2 shows the measured photon dose equivalents for the machines in Vault‐1 and Vault‐2, with the radiation beams pointing downward (gantry angle 180°).

As shown in Fig. 2, the measured values at any point along the maze centerline, with the exception of points less than 1 m from point *A*, are in good agreement with calculated values using an empirical equation following McGinley:^2^

(1) $$D_{\varphi }=5.7\times 10^{-16}\times \varphi_{A}\times 10^{-(d_{2}/6.2)},$$

where $D_{\varphi }$ is the capture gamma dose in Gy per Gy x‐ray dose at isocenter, $d_{2}$ is the distance from point *A* in m, $\varphi_{A}$ is the total neutron fluence at the inner maze entrance, as given by McCall *et al.*,^1^ ^,^ ^9^ and shown here in Eq. (2),

(2) $$\varphi_{A}=\frac{Q_{N}}{4\pi d_{1}^{2}}+\frac{5.4Q_{N}}{2\pi S}+\frac{1.26Q_{N}}{2\pi S},$$

where $\varphi_{A}$ is in units of neutrons per $m^{2}$ per Gy isocentric x‐ray dose, $Q_{N}$ is the apparent neutron source strength in neutrons per Gy isocentric x‐ray dose, and $Q_{N}$ is related to the neutron source strength *Q*; it is equal to *Q* for head shielded with lead, and 0.85*Q* for head shielded with tungsten. With the exception of Vault‐2 and Vault‐7, values of *Q* are from McGinley,^2^ the accelerator heads are shielded by lead, and $Q_{N}$ values are equal to the corresponding *Q* values. The value of $Q_{N}$ for Vault‐2 was measured by the authors and the work is the subject of a separate article. The value of $Q_{N}$ for Vault‐7 was calculated based on data provided by the Radiological Physics Center, Houston, TX. $d_{1}$ is the distance from isocenter to point *A* in meter, *S* is the surface area of the room, excluding the maze, in $m^{2}$.

At points near the inner maze entrances, the photon doses measured are higher than values obtained using Eq. (1). This is because of the contribution of leakage radiation through the accelerator head. The photon doses measured are the combined doses of capture gamma and leakage x‐ray. At points farther down the maze, the direct leakage component and the leakage radiation scattered by the wall become negligibly small compared with the capture gamma. The straight line portions of the plots were used to obtain the tenth value lengths for the two vaults. The average value of 6.2 m is used in the equation. This value is in agreement with the literature.^2^

The coefficient $5.7\times 10^{-16}$ is in units of Gy‐m^2^ per neutron, and was obtained by forcing the calculated values to fit the measured data for $d_{2}>1\;m$ for the two vaults, using the parameters tabulated in Table I.

### B. Neutron dose equivalent

**Figure 3:**
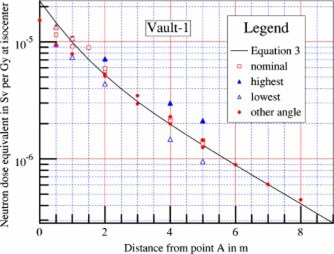
(Color) Neutron dose equivalents measured along the maze (open squares), and calculated using Eq. (3) (solid line). Survey meter anisotropy properties are shown by the difference in readings taken with the meter rotated horizontally. The nominal angle is the angle of the meter at calibration. The highest readings are usually at 45° clockwise from nominal as observed from above the meter (solid triangles). The lowest readings are usually at 225° clockwise from nominal (open triangles). The other angle refers to the readings with the meter rotated 180° from nominal (diamonds). Multiple sets of data point averages taken in different days are displayed in the figure to show data variations.

The measured neutron dose equivalent values along the maze of Vault‐1 are shown in Fig. 3. The collimators were closed to $0\times 0\;{\text{cm}}^{2}$. The radiation beam was pointing downward (gantry angle 180°). The measurements were repeated with the survey meter rotated horizontally at different orientation angles at several points along the maze to evaluate the anisotropy properties. The readings taken with the meter set at the same angle as at calibration are displayed in Fig. 3 and labeled as nominal. The survey meter showed significant anisotropy effect as readings taken at the same point with the meter rotated horizontally were different from nominal. For example, the maximum reading at the 5 m point is 57% higher than nominal, and the minimum reading is 30% below nominal. The differences are less at points in the maze closer to point *A*. It was determined that data taken at nominal angle and at meter rotation angle of 180° (noted in Fig. 3 as “other angle”) are not significant considering experimental errors, with the exception of data points at distances less than 1.5 m from point *A*. At distances close to point *A*, the “nominal” is about the same as the “highest,” and the “other angle” is about the same as the “lowest.” This is most likely due to the fact that at or near point *A*, the general direction of the neutron flow is no longer parallel to the centerline of the maze. For clarity in presentation, data labeled nominal are taken at the same meter rotation angle relative to the centerline of the maze for all points along the maze.

**Figure 4:**
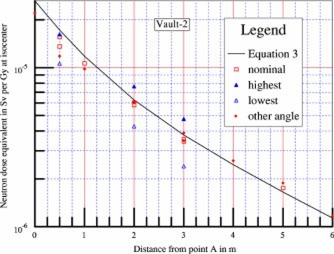
(Color) Neutron dose equivalent along the maze of Vault‐2. Data obtained with the meter orientation the same as at calibration are denoted nominal. The highest readings are usually at 45° clockwise from nominal as observed from above (closed triangle). The lowest readings are usually at 225° clockwise from nominal (open triangle). The other angle refers to the readings with the meter rotated 180° from nominal (diamonds).

Results for Vault‐2 are shown in Fig. 4. Similar anisotropy properties were observed. When the meter was rotated 45° and 225° clockwise from nominal, the neutron readings were usually the highest and the lowest, respectively. At 3 m from point *A*, the highest reading was 39% above, and the lowest reading was 30% below nominal.

**Figure 5:**
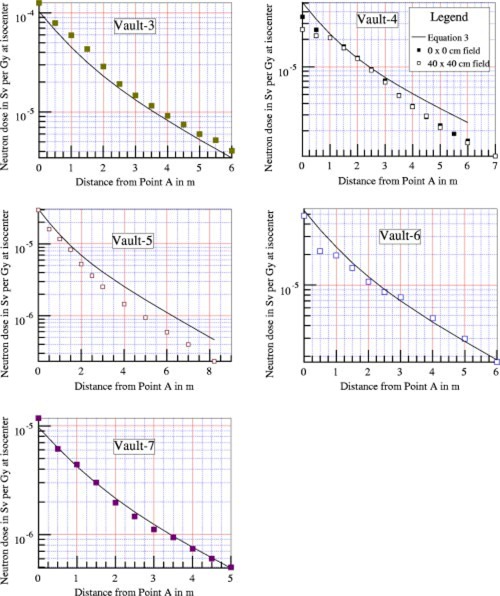
(Color) Neutron dose equivalent measured along the maze for five vaults. The beam is pointing downward, and the field size is $0\times 0\;{\text{cm}}^{2}$ (closed squares) or $40\times 40\;{\text{cm}}^{2}$ (open squares) as noted. Solid lines are calculated values using Eq. (3).

The second set of measurements was made for Vault‐3 to Vault‐7. The vaults are located in different cities in the United States. The measurements were made for routine radiation protection purposes. The radiation beam was pointing downward. The fields sizes were 0×0 or $40\times 40\;{\text{cm}}^{2}$ as noted. The results are shown in Fig. 5.

**Figure 6:**
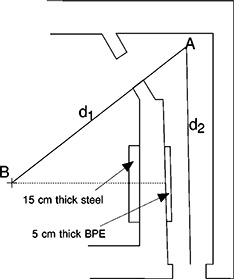
Maze layout of Vault‐5 which shows reduced cross‐sectional area of the inner maze entrance. The 5 cm thick borated polyethylene (BPE) plate installed to reduce the transmitted neutron fluence through the maze wall also serves to reduce the neutron dose along the maze.

Although the measured data for Vault‐5 is within a factor of 2 compared with values predicted by Eq. (3) the deviations are unusually high. Further studies showed that the maze construction of Vault‐5 was modified to reduce the thickness of the maze door. A layer of borated polyethylene (BPE, 5% boron) 5 cm thick and floor to ceiling in height, was installed on the maze side of the maze wall as shown in Fig. 6. The borated BPE has been shown to significantly reduce the thermal neutron fluence.^10^

The empirical equation plotted as solid lines in Figs. 3–5 is

(3) $$D_{n}=2.4\times 10^{-15}\times \varphi_{A}\times \sqrt{\frac{S_{0}}{S_{1}}}\times [1.64\times 10^{-(d_{2}/1.9)}+10^{-(d_{2}/T_{N})}],$$

where $D_{n}$ is the neutron dose equivalent in Sv per x‐ray Gy at isocenter, $\varphi_{A}$ is the total neutron fluence at point *A* in $n-m^{-2}$ per x‐ray Gy at isocenter, as given by Eq. (2), $S_{0}$ is the cross‐sectional area of the inner maze entrance, in $m_{2},\;S_{1}$ is the cross‐sectional area of the maze, in $m_{2},\;d_{2}$ is the distance from the point of measurement to point *A*, in m, and $T_{N}$ is the tenth value length in meter as described below.

The semilog plots of the measured data show that near the inner maze entrance, the neutron dose equivalent decreases with a tenth value length of 1.9 m. This component has been reported by McGinley,^2^ with tenth value length of 1.7 m. The present value of 1.9 m showed better fit for the data presented here. At points farther down the maze, the neutron dose decreases with a longer tenth value length $\left(T_{N}\right)$. Using the straight line portion of the plots, these tenth value lengths were determined for all seven vaults. Attempts were made to correlate the $T_{N}$ with the inner maze entrance cross‐sectional area $S_{0}$, maze cross‐sectional area $S_{1}$, and room surface area *S* for all seven vaults. The study produced a simple equation as follows:

(4) $$T_{N}=2.06\times \sqrt{S_{1}},$$

where $T_{N}$ is the tenth value length in Eq. (3) in m, and $S_{1}$ is the cross‐sectional area of the maze in $m^{2}$.

Similar observations have been reported for thermal neutron attenuation through mazes and ducts (NCRP report 51, Appendix F11).^4^ The tenth value length was found to be proportional to the square root of the product of the height and width of the aperture of the duct or the maze, as was independently found in this work. The coefficients obtained from the curves in NCRP 51 range from 2.5 to 5.5, but the data were mainly presented for purposes of estimating the attenuation along ducts. For mazes of designs studied in this work, the coefficient 2.06 is considered more appropriate.

With the values of $T_{N}$ determined as above, the neutron dose data were further analyzed. Attempts were made to correlate the data for all seven vaults with the ratio $\left(S_{0}/S_{1}\right)$, simple exponents of the ratio, as well as $S_{0}$ and $S_{1}$ separately. Kersey^3^ reported that the dose is directly related to the ratio $\left(S_{0}/S_{1}\right)$. However, these data do not support such a relationship. Using semilog graphical plots, with more weight given to the data for Vault‐1 and Vault‐2, it was found that the square root of the ratio would give better agreements.

The coefficient $2.4\times 10^{-15}$ is in units of Sv‐m^2^ per neutron, and was obtained by forcing the calculated value to agree with the measured data for $d_{2}\;=2\;m$ for Vault‐1 and Vault‐2, using the parameters tabulated in Table I. Plots using the empirical Eq. (3) are shown in Figs. 3–3;5, showing good agreement with the experimental data for the other five vaults.

### C. Neutron dose with collimators fully opened

**Figure 7:**
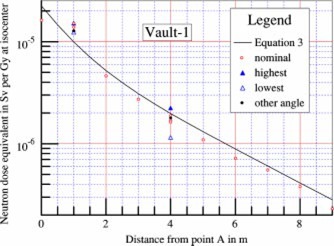
(Color) Neutron dose equivalents measured with the collimators opened to the maximum field size, $40\times 40\;{\text{cm}}^{2}$ (circles). The radiation is pointing downward (Gantry angle 180°), intercepted by a solid phantom. The solid line is the neutron dose for $0\times 0\;{\text{cm}}^{2}$ field size based on Eq. (3). Multiple data point averages taken at 1 and 4 m are displayed in the figure to show data variations excluding meter anisotropy Meter anisotropy is shown by the data points marked by the triangles and diamonds as described in the caption of Fig. 3.

Neutron dose data were obtained at several points along the centerline of the maze of Vault‐1, with the collimator settings varied. The radiation beam was set pointing downward, with a $40\times 40\times 40\;{\text{cm}}^{3}$ solid phantom placed at the isocenter at 100 cm source‐surface distance. The neutron dose in the maze was found to be lower when the collimators were set to larger field sizes. This observation has been reported by McGinley and Huffman.^11^ Comparing with the neutron dose data for $0\times 0\;{\text{cm}}^{2}$ field size with no phantom, the $40\times 40\;{\text{cm}}^{2}$ data are about 10% lower for the accelerator and vault configuration used in this study. However, at or near the inner maze entrance, the effect of collimator setting is reversed. The neutron dose increases with the increase in field size. The $40\times 40\;{\text{cm}}^{2}$ value is about 23% higher than that of $0\times 0\;{\text{cm}}^{2}$ field size for Vault‐1 at 1 m from point *A*. These observations are best illustrated by Fig. 7.

### D. Maze neutron dose at various gantry angles

**Figure 8:**
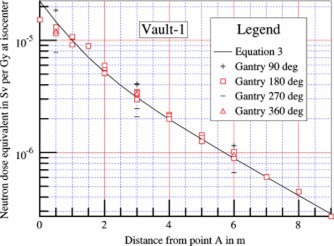
(Color) Dependence of maze neutron dose on the gantry angle of the accelerator, at 0.5, 3, and 6 m from point *A*. The collimators are closed. The solid line is for gantry angle 180° (beam pointing downward) calculated using Eq. (3).

The effect of gantry angle on neutron dose in the maze was studied using the 15 MV photon beam of the accelerator in Vault‐1. Measurements were made with the neutron meter oriented at the nominal angle described above, at three chosen locations along the centerline of the maze. As shown in the following figure, when the gantry was rotated to 90° (radiation beam pointing from *B* to *C* in Fig. 1), the maze neutron dose equivalent was highest. The proximity of the accelerator head, which was the source of neutrons, to the inner maze entrance resulted in higher readings. At 270° (radiation beam pointing in the *C* towards *B* direction shown in Fig. 1), the neutron dose was lowest, as the head of the accelerator was farthest away. Similar observations have been reported by McGinley and Huffman.^11^ At gantry angles of 180° and 360°, the doses in the maze were in between, but slightly higher than the average of the highest and lowest readings. The dose equivalents at 90° gantry angle were higher than those at 270° by a factor of 1.7 to 2.4 for the three measurement points ($d_{2}=0.5$, 3, and 6 m), as shown in Fig. 8.

## CONCLUSIONS

In this work the neutron and photon dose equivalents have been measured at various points along the centerlines of the mazes for several accelerator vaults with a design similar to that shown in Fig. 1. Based on the measured data for two of the vaults, the capture gamma dose has been related to the total neutron fluence at the inner maze entrance by Eq. (2). The dose decreases exponentially with the increase in distance $\left(d_{2}\right)$ from point *A*, with a tenth value length of 6.2 m, according to Eq. (1). Based on the measured data for all seven vaults, the neutron dose equivalents at any point along the maze have been related to the total neutron fluence at the inner maze entrance. The neutron dose decreases exponentially with $d_{2}$ as the sum of two components. The first component has a tenth value length of 1.9 m, and the second component has a tenth value length of $T_{N}$. The value of $T_{N}$ is proportional to the square root of the cross‐sectional area of the maze, as given by Eq. (4). The neutron dose has been found to be proportional to the square root of the ratio of the cross‐sectional areas of the inner maze entrance and the maze, as given by Eq. (3). Both Eqs. (1) and (3) are fitted for mazes of length up to 9 m. Using the equations and other factors available in the literature, one can obtain the capture gamma and neutron doses in a maze for an accelerator vault of similar design. The equations are for radiation beams pointing downward with the collimators closed. When the collimators are opened, the neutron dose equivalents in the maze are lower, except near the inner maze entrance. However, the higher measured value near the inner maze entrance may be due to the difference in neutron spectrum and the nonlinear response of the neutron meter. This is under further study. The neutron dose equivalent in the maze is highest when the gantry is rotated to the position where the head of the accelerator is closest to the inner maze entrance. As presented above, the empirical equations are able to give capture gamma and neutron doses within a factor of 2 of the measured values. However, the vaults included in the study have relatively similar cross‐sectional areas of the mazes and inner maze entrances. The agreements are worst for the two vaults with $S_{0}$ values at both ends of the range. Caution should be given when applying the equations for vaults of significantly different designs. The effect of smaller $S_{0}$ on the reduction of maze neutron dose merits the extra effort of vault designers to find ways to minimize the area of the inner maze entrance. Specifically, the height of the entrance should not be more than necessary, such as for the rigging in of the accelerator. Furthermore, the neutron source strengths used in the equations were measured for recent model accelerators. Older models are known to have higher *Q* values.^12^ ^,^ ^13^ Finally, the capture gamma dose in the maze may be related to the cross‐sectional areas of the inner maze opening and the maze. This is being studied.
